# Supplementary material for: Fine Mapping without Phenotyping: Identification of Selection Targets in Secondary Evolve and Resequence Experiments
Source: Genome Biol Evol. 2021 Jun 30;13(8):evab154. doi: 10.1093/gbe/evab154 (PMC8358229; doi:10.1093/gbe/evab154)
Supplement: evab154_Supplementary_Data [file evab154_supplementary_data.pdf]

Supplementary Figures

Figure S1. Simulated versions of the 1:1 crossing scheme.

Figure S2. Number of ties in the 1:1_1f and dil:mt crossing schemes.

Figure S3. Power of the 1:1_1f and dil:mt crossing schemes after 20 generations of adaptation.

Figure S4. Resolution of the 1:1_1f and dil:mt crossing schemes after 20 generations of adaptation.

Figure S5. Allele frequency trajectories of selected SNPs in 1:1_2f.

Figure S6. Allele frequency trajectories of focal SNPs in 5 different crossing schemes.

Figure S7. Number of ties in 5 different crossing schemes.

Figure S8. Power of 5 different crossing schemes with 30 replicates per simulation.

Figure S9. Resolution of 5 different crossing schemes with 30 replicates per simulation.

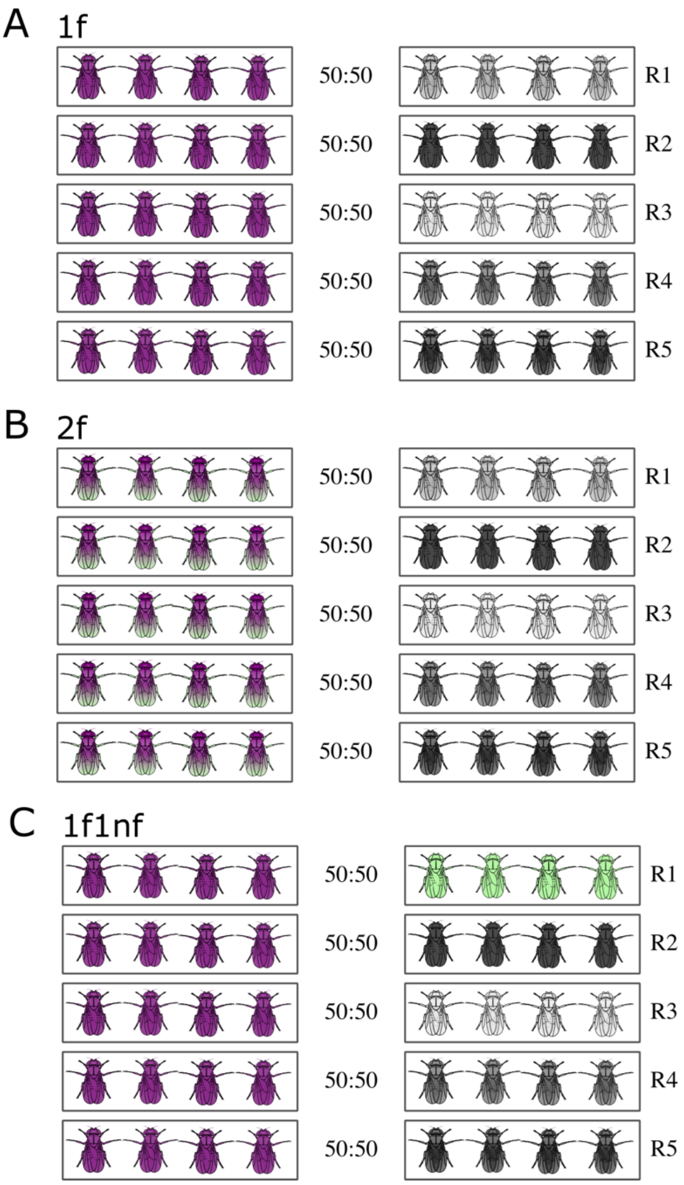

Figure S1. Simulated versions of the 1:1 crossing scheme. (A) Default version of the 1:1 crossing scheme (=**1f** for 1 focal SNP). Inbred flies with one target of selection (purple) are crossed to inbred flies without known beneficial variants. The starting frequency of each genotype is 50%. In each replicate the line with the beneficial allele (focal line, purple) is crossed to a different line lacking beneficial mutations (non-focal lines are colored in different shades of grey). Figure S1A is equal to Figure 1B in the main manuscript. (B) 2 focal SNPs (=**2f**) version of the 1:1 crossing scheme. The focal line carries two beneficial variants: the focal SNP we aim to fine map (purple) and one additional target of selection (green). (C) 1 focal, 1 non-focal SNP (=**1f1nf**) version of the 1:1 crossing scheme: One non-focal line carries on additional beneficial variant (green).

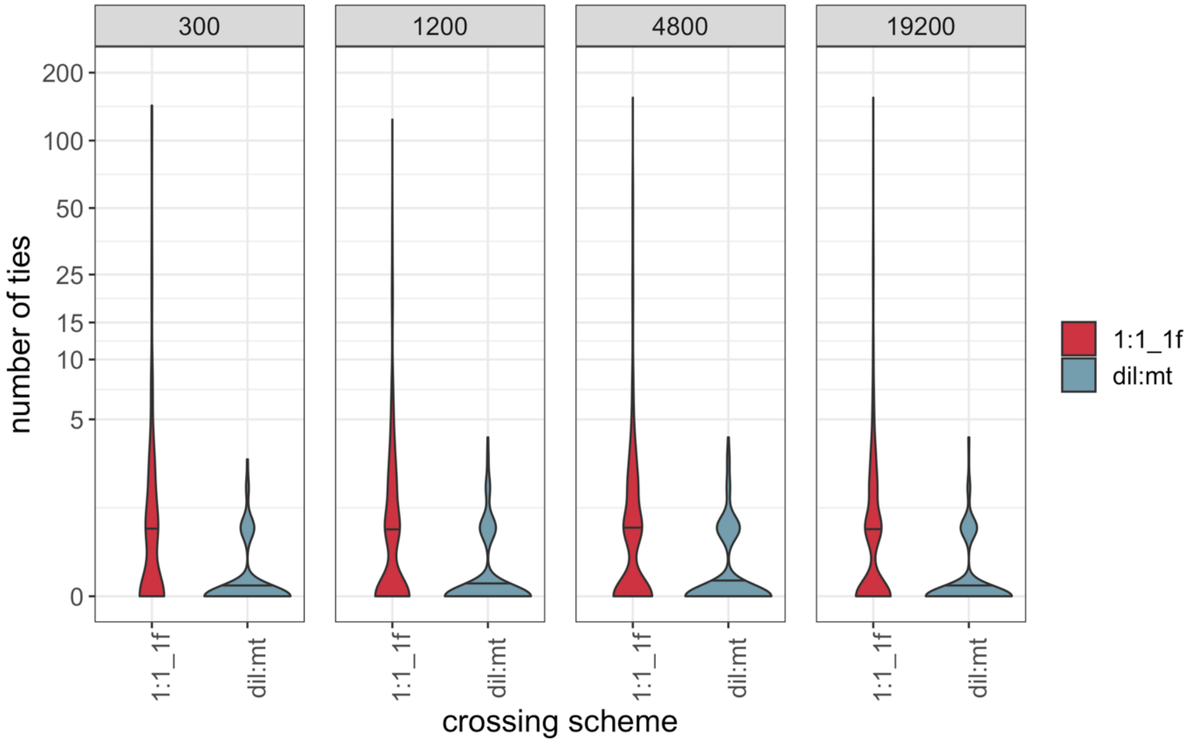

Figure S2. Violin plots of the number of ties (y axis = $\log_{10}$ (number of ties + 1)) for simulations where the true target of selection has the highest Cochran-Mantel-Haenszel (CMH) test statistic (success-A). The black horizontal lines in the violin plots display the median number of observed ties. Each panel shows the results for one particular simulated population size. Ties are defined as neighboring SNPs that have the same CMH test statistic as the target of selection.

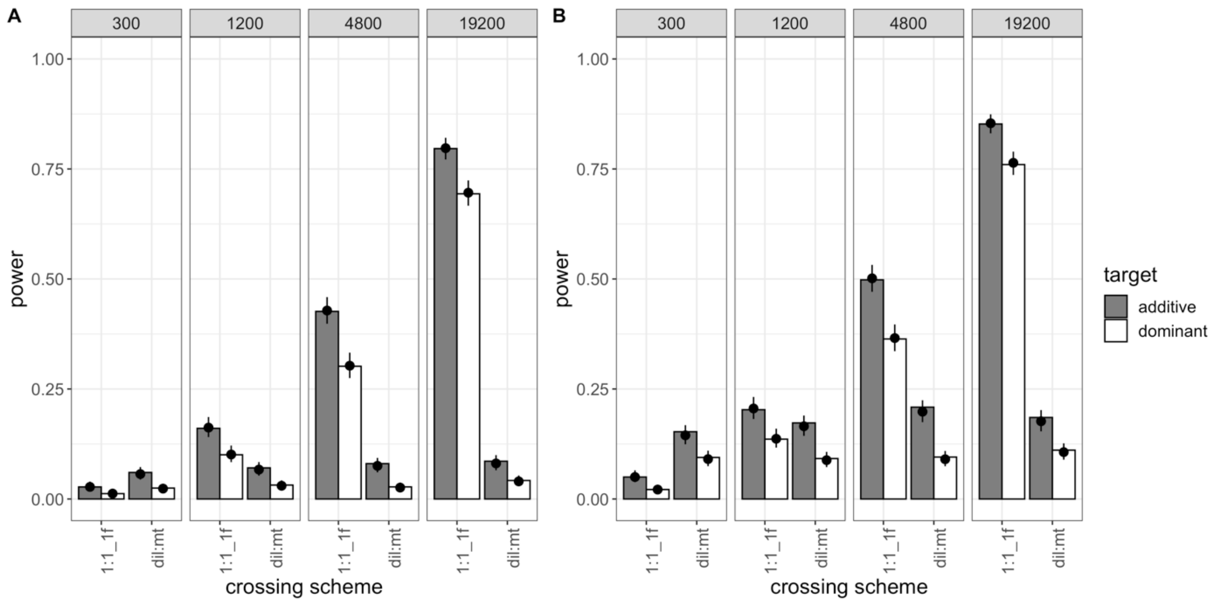

Figure S3. Power of the 1:1_1f and dil:mt crossing scheme at different population sizes (2,000 simulations/experimental design) after 20 generations of adaptation. Bars show the power (i.e., proportion of successful simulations) separately for each combination of crossing scheme (1:1_1f, dil:mt), population size (300; 1,200; 4,800; 19,200 individuals), and dominance coefficient (additive in grey, dominant in white). The dots with error bars display the model fit (Model 1) and its 95 % confidence interval. For the model fit, the selection coefficient was fixed to its global average, and combination-specific average starting allele frequencies were used. (A) shows the results for success-A (= selection target is the SNP with the highest Cochran-Mantel-Haenszel (CMH) test statistic) (B) shows the results for success-B (= selection target is not more than 100 SNPs away from the SNP with the highest CMH test statistic).

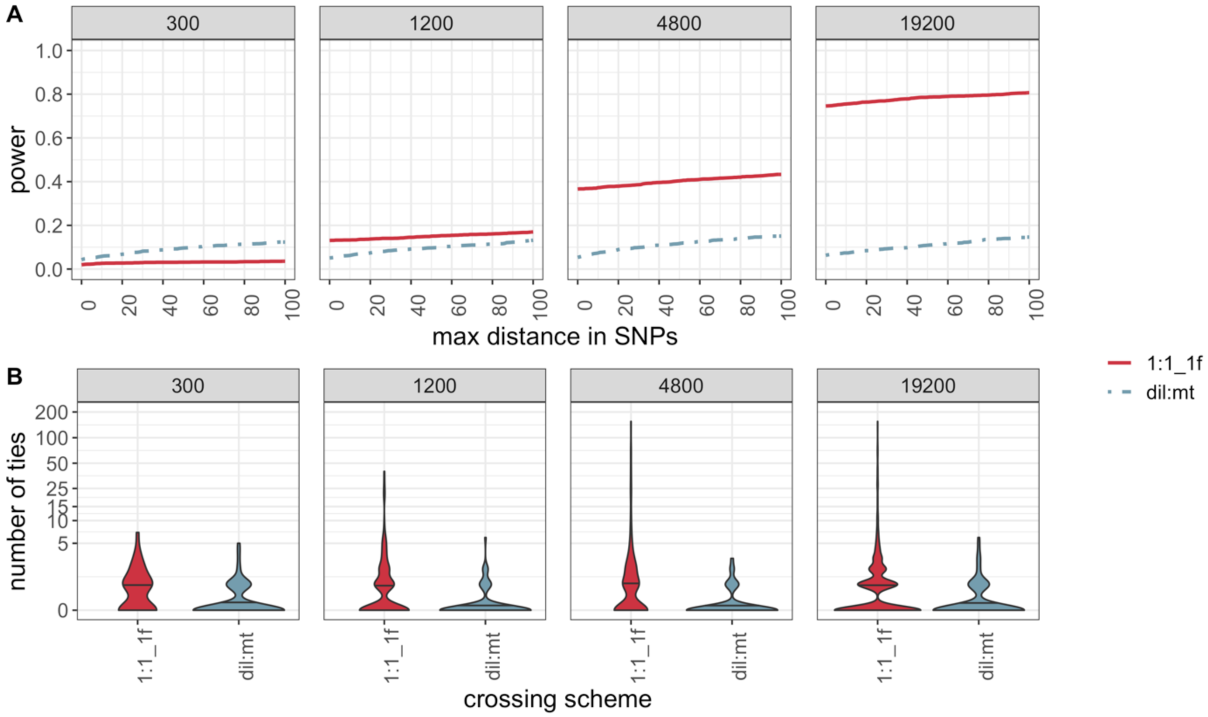

Figure S4. Resolution for the 1:1_1f and dil:mt crossing scheme simulated with different population sizes after 20 generations of adaptation. (A) Proportion of simulations (y-axis) that do not exceed a maximum distance in SNPs (x-axis) between the SNP with the highest Cochran-Mantel-Haenszel (CMH) test statistic and the true target of selection (B) Violin plots of the number of ties (y axis = log10 (number of ties + 1)) for simulations where the true target of selection has the highest CMH test statistic (success-A). The black horizontal lines in the violin plots display the median number of observed ties. Ties are defined as neighboring SNPs that have the same CMH test statistic as the target of selection.

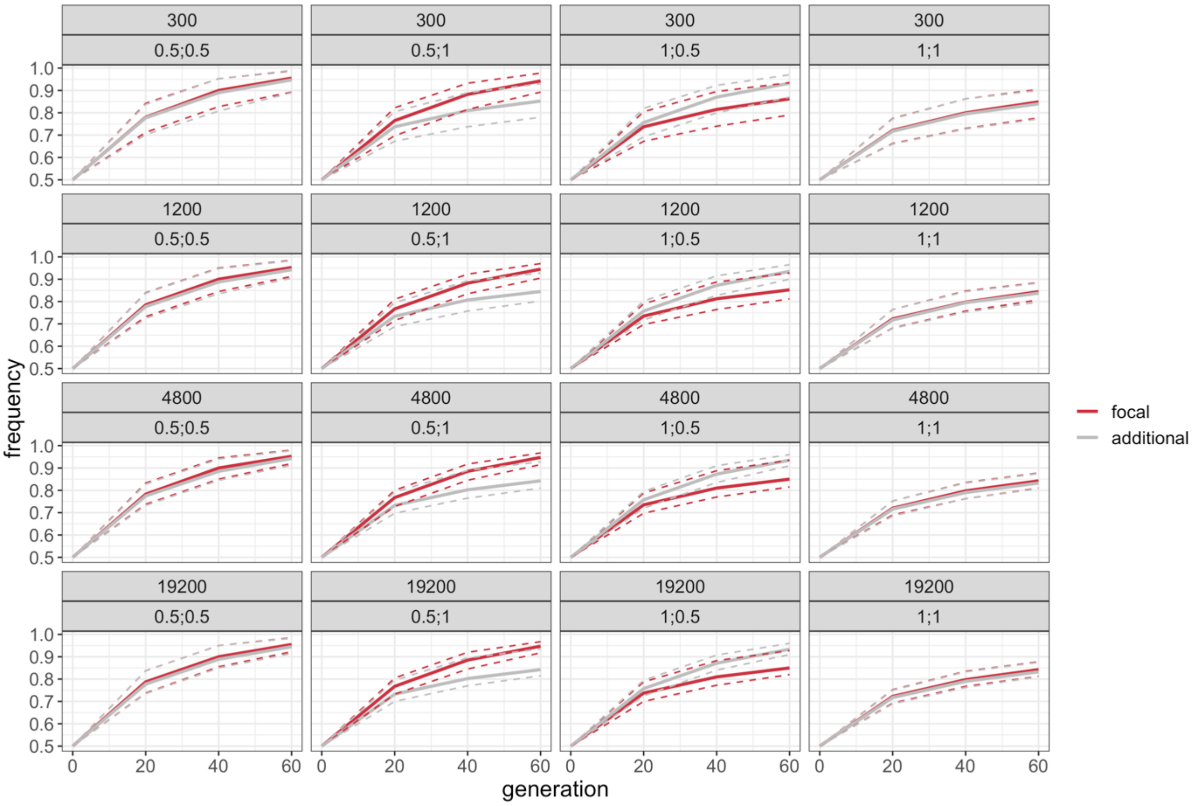

Figure S5. Median allele frequency trajectory of focal (red) and additional targets (grey) in 1:1_2f. For each single simulation, the target frequencies of a distinct generation were averaged over five replicates. Solid lines show the median allele frequency trajectory over 2,000 independent simulations per experimental design. Dashed lines show the 5 and 95 percentiles of the allele frequency trajectories. Each panel shows the median allele frequency trajectory of one particular dominance coefficient (focal target; additional target) and population size (300; 1,200; 4,800; 19,200 individuals) combination.

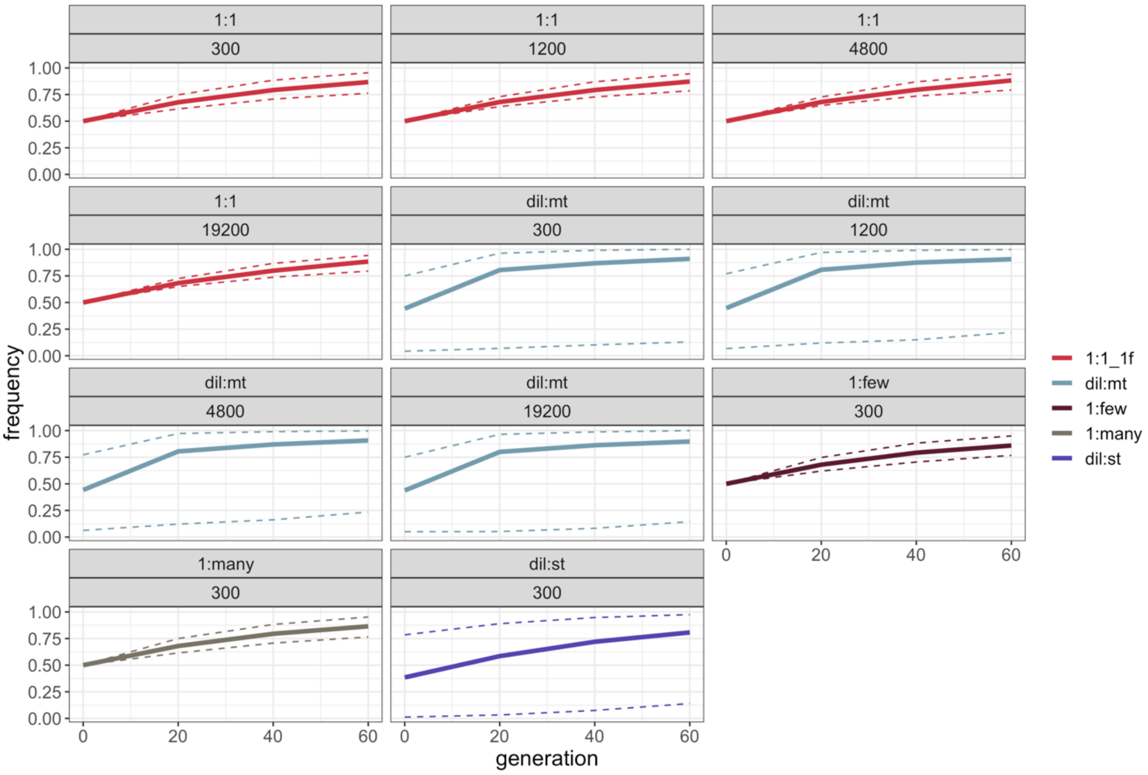

Figure S6. Median allele frequency trajectory of focal targets in different experimental designs. For each single simulation, the focal target frequency of a distinct generation was averaged over five replicates. Solid lines show the median allele frequency trajectory over 2,000 independent simulations per experimental design. Dashed lines show the 5 and 95 percentiles of the allele frequency trajectories. Each panel shows the median allele frequency trajectory of one particular crossing scheme (1:1_1f, dil:mt, 1:few, 1:many, dil:st) and population size (300; 1,200; 4,800; 19,200 individuals) combination.

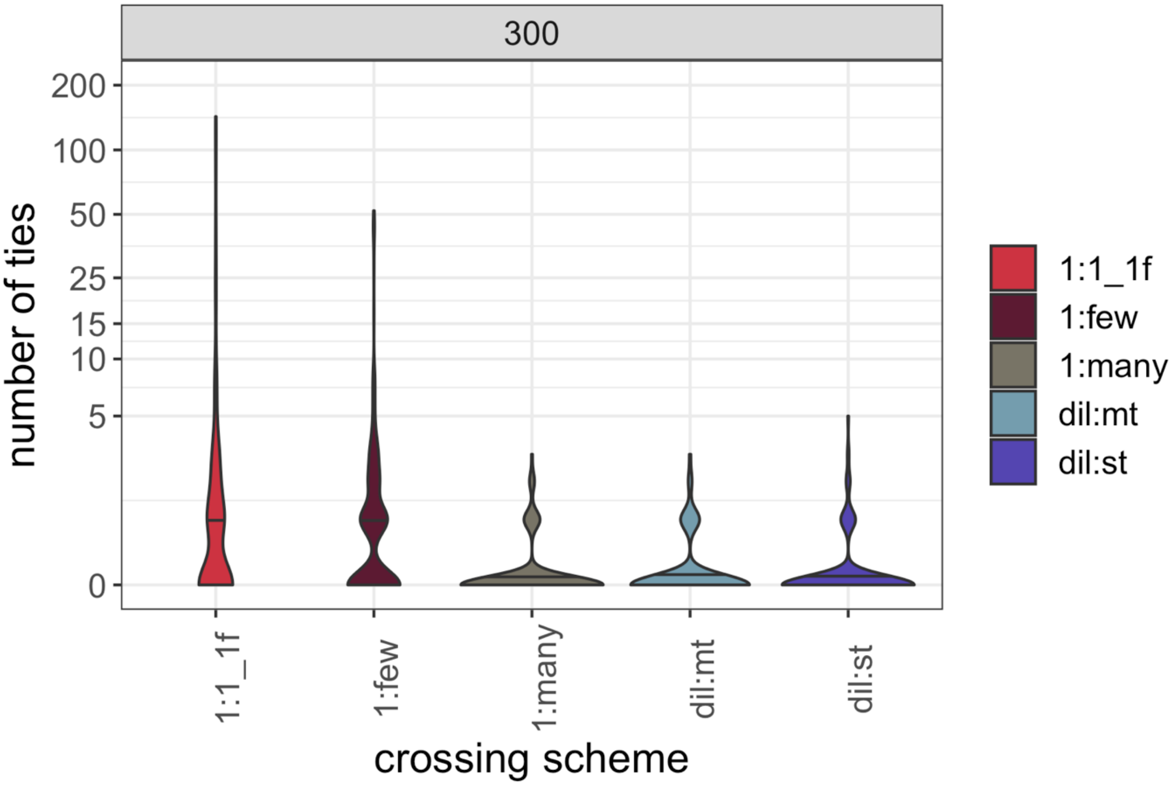

Figure S7. Violin plots of the number of ties (y axis = log10 (number of ties + 1)) for simulations where the true target of selection has the highest Cochran-Mantel-Haenszel (CMH) test statistic (success-A, population size = 300 individuals; 5 replicates; 2,000 simulations/experimental design). The black horizontal lines in the violin plots display the median number of observed ties. Ties are defined as neighboring SNPs that have the same CMH test statistic as the target of selection.

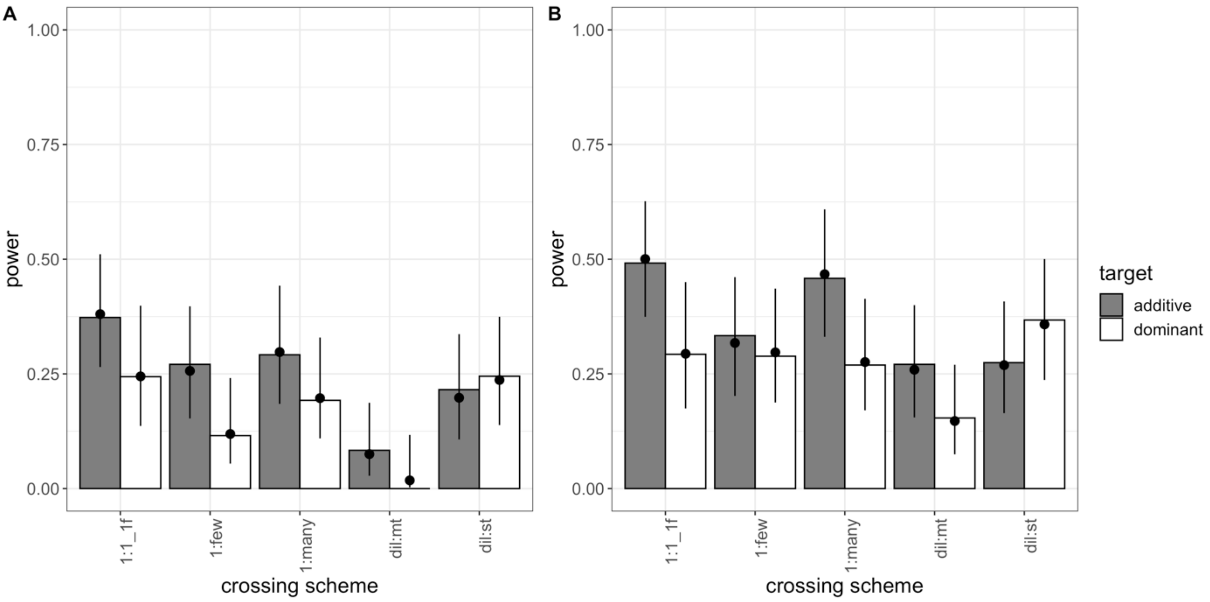

Figure S8. Power of five different crossing schemes (population size = 300 individuals; 30 replicates; 100 simulations/experimental design). Bars show the power (i.e., proportion of successful simulations) separately for each combination of crossing scheme and dominance coefficient (additive in grey; dominant in white). The dots with error bars display the estimate from the fitted model (Model 3) and its 95 % confidence interval. For the model fit, the selection coefficient was fixed to its global average, and combination-specific average starting allele frequencies were used. (A) shows the results for success-A (= selection target is the SNP with the highest Cochran-mantel-Haenszel (CMH) test statistic), (B) shows the results for success-B (= selection target is not more than 100 SNPs away from the SNP with the highest CMH test statistic).

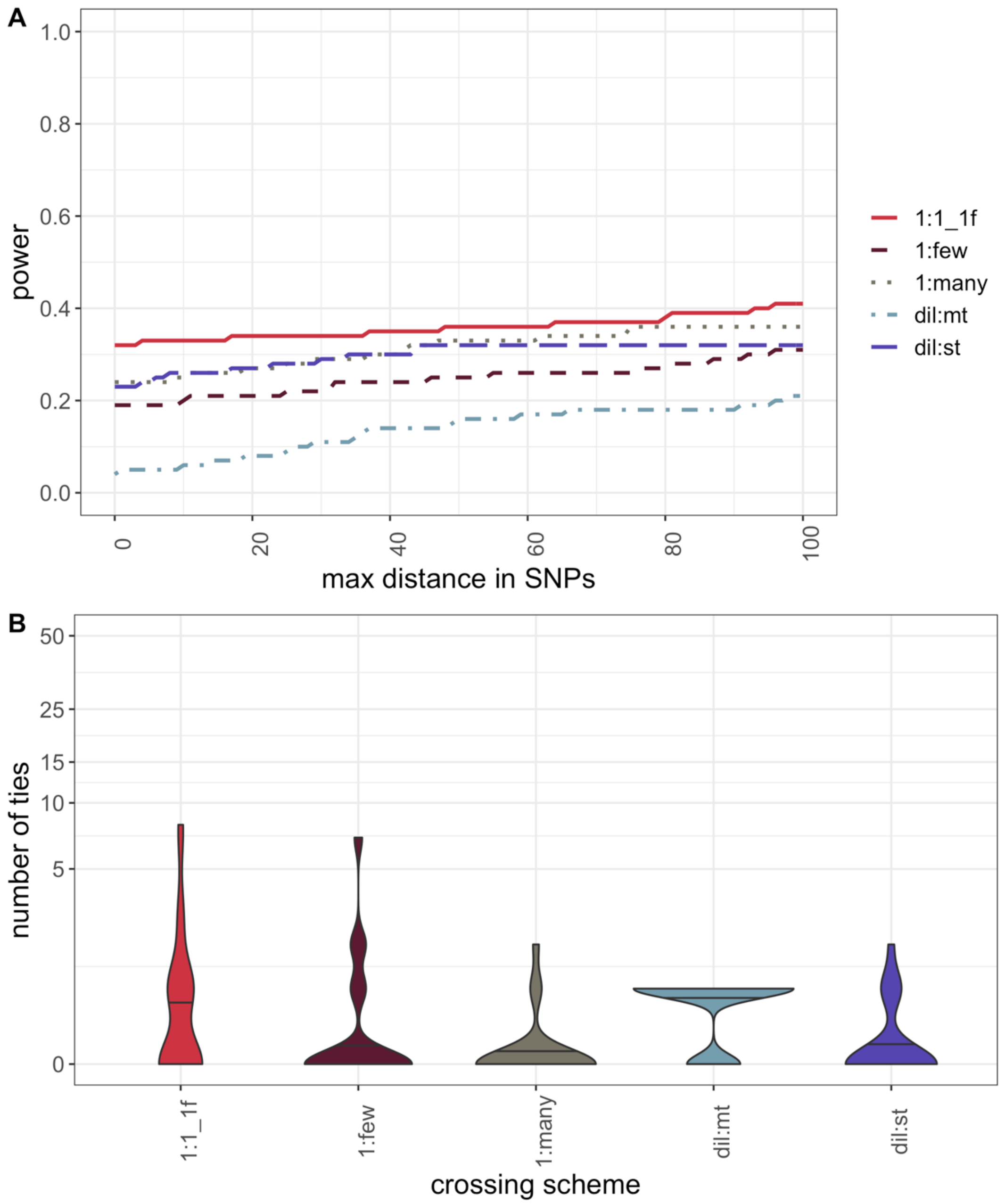

Figure S9. Resolution for five different crossing schemes (population size = 300 individuals; 30 replicates; 100 simulations/experimental design). (A) Proportion of simulations (y-axis) that do not exceed a maximum distance in SNPs (x-axis) between the SNP with the highest Cochran-Mantel-Haenszel (CMH) test statistic and the true target of selection (B) Violin plots of the number of ties (y axis = $\log_{10}$ (number of ties + 1)) for simulations where the true target of selection has the highest CMH test statistic (success-A). The black horizontal lines in the violin plots display the median number of observed ties. Ties are defined as neighboring SNPs that have the same CMH test statistic as the target of selection.

Supplementary Tables

Table S1. Number of SNPs per crossing scheme.

Table S2. Type II ANOVA (Model 1).

Table S3. Nagelkerke's R^2-index (Model 1).

Table S4. Type II ANOVA (Model 2).

Table S5. Nagelkerke's R^2-index (Model 2).

Table S6. Type II ANOVA (Model 3).

Table S7. Nagelkerke's R^2-index (Model 3).

Table S1. Number of SNPs per crossing scheme on chromosome-arm 2L.

crossing scheme	median	range
1:1	523,008	479,507 – 527,771
1:few	526,510	522,470 – 530,230
1:many	970,466	970,466 – 970,466
dil:mt	970,466	970,466 – 970,466
dil:st	970,466	970,466 – 970,466

Table S2. Type II ANOVA of explanatory variables for the analysis of success in secondary Evolve and Resequence studies with model 1 after 60 and 20 generations of adaptation. cross= crossing scheme; h= dominance coefficient; s= selection coefficient; af= mean starting allele frequency of the focal target over 5 replicates; N= population size; cross:h = interaction term between crossing scheme and dominance coefficient; h:af = interaction term between dominance coefficient and mean starting allele frequency; cross:N = interaction term between crossing scheme and population size ; h:N = interaction term between dominance coefficient and population size; cross:h:N = interaction term between crossing scheme, dominance coefficient, and population size.

**generations**	**term**	**df**	χ^2	***P***	χ^2	***P***
			**success-A**		**success-B**	
60	cross*	1	3188.7	<0.001	2514.70	<0.001
	h*	1	687.5	<0.001	652.41	<0.001
	s [1]	1	67.4	<0.001	73.39	<0.001
	af*[1]	1	3.9	0.048	0.69	0.407
	N*	3	1791.3	<0.001	1898.77	<0.001
	cross:h	1	2.1	0.145	8.24	0.004
	h:af	1	18.5	<0.001	16.67	<0.001
	cross:N	3	315.5	<0.001	677.98	<0.001
	h:N	3	6.8	0.079	2.70	0.440
	cross:h:N	3	3.8	0.286	16.12	0.001
20	cross*	1	2079.94	<0.001	1056.48	<0.001
	h*	1	139.04	<0.001	181.36	<0.001
	s [1]	1	18.21	<0.001	58.96	<0.001
	af* [1]	1	39.91	<0.001	77.13	<0.001
	N*	3	2589.34	<0.001	2204.36	<0.001
	cross:h	1	3.73	0.053	1.15	0.284
	h:af	1	0.02	0.898	0.36	0.551
	cross:N	3	573.81	<0.001	1156.81	<0.001
	h:N	3	0.58	0.900	0.87	0.833
	cross:h:N	3	1.29	0.732	4.68	0.197

* In type II ANOVA, main effects are not corrected for the interaction part of the model, while interaction terms are corrected for the main effects in the model.

[1] multiplied by 100, and z-transformed to mean = 0, and sd =1; Mean/standard deviation of the original value was 0.463/0.130 for the starting allele frequency, and 0.086/0.009 for the selection coefficient respectively

Table S3. Nagelkerke's R^2-index of each explanatory variable including all its interactions in the logistic regression (Model 1). If the reduced model explains the data as well as a full model that includes the evaluated effect, Nagelkerke's R^2-index is 0.

**generations**	**effect**	**R^2 success-A**	**R^2 success-B**
60	crossing scheme	0.305	0.264
	dominance coefficient	0.077	0.069
	selection coefficient	0.007	0.007
	mean starting allele frequency	0.003	0.002
	population size	0.204	0.224
20	crossing scheme	0.283	0.211
	dominance coefficient	0.019	0.021
	selection coefficient	0.002	0.007
	mean starting allele frequency	0.005	0.009
	population size	0.322	0.295

Table S4. Type II ANOVA of explanatory variables for the analysis of success in secondary Evolve and Resequence studies with model 2. architecture= version of the 1:1 crossing scheme in combination with the dominance coefficient of the additional target; h= dominance coefficient of the selection target of interest; s= selection coefficient; N= population size; architecture:h = interaction term between architecture and dominance coefficient of the selection target of interest; architecture:N = interaction term between architecture and population size ; h:N = interaction term between dominance coefficient of the selection target of interest and population size; architecture:h:N = interaction term between architecture, dominance coefficient of the selection target of interest, and population size.

**term**	**df**	χ^2	***P***	χ^2	***P***
		**success-A**		**success-B**	
architecture*	4	855.4	<0.001	1509.9	<0.001
h*	1	2742.4	<0.001	2755.9	<0.001
s [1]	1	181.9	<0.001	137.5	<0.001
N*	3	5230.6	<0.001	6661.9	<0.001
architecture:h	4	697.8	<0.001	708.6	<0.001
architecture:N	12	17.5	0.132	42.7	<0.001
h:N	3	12.3	0.006	3.0	0.399
architecture:h:N	12	27.9	0.006	29.8	0.003

* In type II ANOVA, main effects are not corrected for the interaction part of the model, while interaction terms are corrected for the main effects in the model.

[1] multiplied by 100, and z-transformed to mean = 0, and sd =1; Mean/standard deviation of the original value was 0.085/0.009.

Table S5. Nagelkerke's R^2-index of each explanatory variable including all its interactions in the logistic regression (Model 2). If the reduced model explains the data as well as a full model that includes the evaluated effect, Nagelkerke's R^2-index is 0.

**effect**	**R^2 success-A**	**R^2 success-B**
architecture	0.107	0.158
dominance coefficient	0.201	0.210
selection coefficient	0.012	0.009
population size	0.285	0.348

Table S6. Type II ANOVA of explanatory variables for the analysis of success in secondary Evolve and Resequence studies with model 3. cross= crossing scheme; h= dominance coefficient; s= selection coefficient; af= mean starting allele frequency of the focal target over 5 replicates; cross:h= interaction term between crossing scheme and dominance coefficient; h:af= interaction term between dominance coefficient and mean starting allele frequency.

**generations**	**term**	**df**	χ^2	***P***	χ^2	***P***
			**success-A**		**success-B**	
5	cross*	4	218.65	<0.001	93.01	<0.001
	h*	1	371.35	<0.001	321.13	<0.001
	s [1]	1	76.20	<0.001	72.09	<0.001
	af* [1]	1	5.55	0.019	12.66	<0.001
	cross:h	4	77.19	<0.001	97.21	<0.001
	h:af	1	7.56	0.006	7.07	0.008
30	cross*	4	28.79	<0.001	11.28	0.024
	h*	1	5.13	0.024	4.40	0.036
	s [1]	1	2.63	0.105	3.65	0.056
	af* [1]	1	1.34	0.247	0.13	0.716
	cross:h	4	2.68	0.612	5.41	0.247
	h:af	1	1.73	0.189	1.01	0.315

* In type II ANOVA, main effects are not corrected for the interaction part of the model, while interaction terms are corrected for the main effects in the model.

[1] multiplied by 100, and z-transformed to mean = 0, and sd =1; Mean/standard deviation of the original value were 0.463/0.133 for the starting allele frequency, and 0.085/0.009 for the selection coefficient respectively.

Table S7. Nagelkerke's R^2-index of each explanatory variable including all its interactions in the logistic regression (Model 3). If the reduced model explains the data equally well as a full model that includes the evaluated effect, Nagelkerke's R^2-index is 0.

**replicates**	**effect**	**R^2 success-A**	**R^2 success-B**
5	crossing scheme	0.061	0.033
	dominance	0.095	0.076
	selection coefficient	0.016	0.013
	starting allele frequency	0.003	0.004
30	crossing scheme	0.097	0.046
	dominance	0.034	0.034
	selection coefficient	0.009	0.010
	starting allele frequency	0.010	0.003
